# Etymologia: *Emmonsia*

**DOI:** 10.3201/eid2302.ET2302

**Published:** 2017-02

**Authors:** Ronnie Henry

**Keywords:** Emmonsia, fungi, fungal infections, adiaspiromycosis, spores, pulmonary disease, polyphyletic, Chester W. Emmons

## *Emmonsia* [ĕ-monʹse-ə]

*Emmonsia* (Figure) is a genus of soil fungus that can cause adiaspiromycosis, a pulmonary disease common in wild animals, but rare in humans, as well as disseminated disease. When aerosolized spores are inhaled, they enlarge dramatically, from 2–4 μm to 40–500 μm in diameter. Because these swollen cells do not replicate, Emmons and Jellison termed them “adiaspores” (from the Greek *a* [“not”] + *dia* [“by”] + *spora* [“sowing”]). *Emmonsia* was first described by Chester W. Emmons, senior mycologist with the US Public Health Service, as *Haplosporangium parvum* in 1942. In 1958, it was reclassified into a separate genus and named in honor of Emmons. Recent phylogenetic analyses have concluded that fungi in this genus are polyphyletic, and proposed taxonomic changes may render the genus name obsolete.

**Figure:**
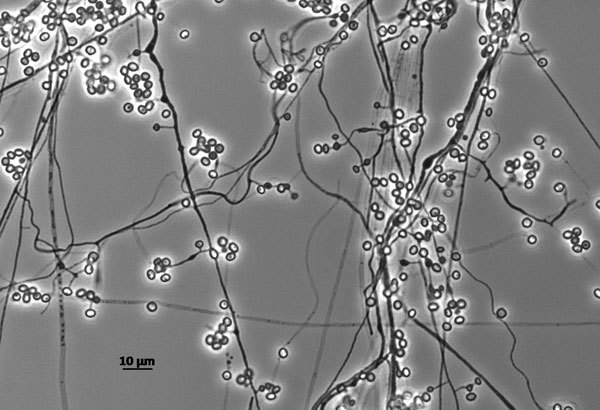
Isolate UAMH 125 *Emmonsia parva* grown in slide culture preparation for 14 days at 25°C. Image courtesy of Lynne Sigler, University of Alberta Microfungus Collection (now UAMH Centre for Global Microfungal Biodiversity, https://www.uamh.ca), University of Alberta, Edmonton, Alberta, Canada.
